# The McGill Quality of Life Questionnaire-Revised (MQOL-R). Psychometric properties and validation of a Brazilian version on palliative care patients: a cross-sectional study

**DOI:** 10.1186/s12955-020-01621-8

**Published:** 2020-11-14

**Authors:** Paul Vicuña Serrano, Gerardo Beltran Serrano, Iraci L. S. Torres, Roberta Rossi Graudner, Wolnei Caumo

**Affiliations:** 1 Post-Graduate Program in Medical Sciences, School of Medicine, Universidade Federal Do Rio Grande Do Sul (UFRGS), Ramiro Barcelos, 2350, Bairro Rio, Porto Alegre, CEP 90035-003 Brazil; 2 Laboratory of Pain and Neuromodulation At Hospital de Clínicas de Porto Alegre (HCPA), Porto Alegre, RS Brazil; 3 Pain and Palliative Care At Hospital de Clínicas de Porto Alegre (HCPA), Porto Alegre, Brazil; 4 School of Medicine - Universidade Federal do Rio Grande do Sul. Coordinator of the Center of Pain Pharmacology and Neuromodulation: Pre-clinical Researches LAFDOR., Porto Alegre, Brazil; 5 School of Medicine, Universidade Federal Do Rio Grande Do Sul (UFRGS), Porto Alegre, Brazil; 6 Psychology Department, Universidad Católica de Cuenca, UCACUE, Cuenca, Ecuador

**Keywords:** Quality of life, Palliative care patients, Karnofsky performance, MQOL-R

## Abstract

**Background:**

To assess the psychometric properties, including internal consistency, construct validity, criterion validity, criterion-group validity, and responsiveness, the Reviewed McGill Quality of Life Questionnaire (MQOL-R), into Brazilian Portuguese-(BrP). Also, to analyze the relationship of the BrP-MQOL-R with the scores on the Karnofsky Performance Scale (KPS) and on the Numerical Pain Scale (NPS 0–10).

**Methods:**

The BrP-MQOL-R was administered to a sample of 146 adults (men = 78). A team of experts translated the MQOL-R according to international guidelines. Convergent validity and Confirmatory factor analysis (CFA) was performed.

**Results:**

The BrP-MQOL-R Cronbach’s alpha was 0.85. CFA supported the original four-factor structure, with the following revised model fit-indices: PCLOSE = 0.131, Tucker-Lewis Index (TLI) rho 2 = 0.918, incremental fit index (IFI) delta 2 = 0.936. The convergence validity is supported by a significant correlation between BrP-MQOL-R total scores and their subscales with KPS and with the single item related to the quality of life. And by a converse correlation with the pain scores in the NPS (0–10). Receiver operator characteristics (ROC) analysis showed subjects with KPS equal to or lower than 30% could be discriminated from those with scores on KPS higher than 30% by an area under the curve (AUC) = 0.71, sensitivity = 97%, and specificity = 92%).

**Conclusion:**

The BrP-MQOL-R proves to be a reliable instrument for assessing the quality of life (QOL) in palliative care (PC), with primary evidence of validity. BrP-MQOL-R presented adequate discriminate properties to identify distinct conditions that impact the QOL in PC.

## Background

Palliative care is specialized medical care for people living with a severe illness with a life-threatening disease to prevent and relieve suffering through early identification, with appropriate evaluation and treatment of pain, physical, psychosocial, and spiritual problems. The palliative care provides relief from the symptoms to reduce the stress of the illness coming to the comfort of suffering and improvement of the Quality of Life (QOL) for both the patient and the family” [1]. The QOL comprises physical, emotional, psychological, and social dimensions. The concept must be contextualized since the term “quality of life” can have different meanings. The definition of QOL refers to patients' well-being with a terminal disease, including the dimensions mentioned above. Accordingly, the McGill Quality of Life Questionnaire (MQOL) was developed to measure the QOL of people at the end of life, to overcome specific limitations with the QOL measures existing: (i) They were too long for palliative care patients. (ii) They did not assess the existence or spiritual well-being. (iii) They focused exclusively on negative aspects of QOL, even if positive and negative factors influenced the quality of life [2–5]. Thus, the MQOL was developed with particular interest to assess physical symptoms (Physical Wellbeing; Physical Symptoms) that allow a brief symptom measurement as the Edmonton Symptom Assessment System (ESAS) [6, 7]. According to an earlier study, physical symptoms are essential predictors on measuring global QOL, and they have been of great importance to people with life-threatening illnesses [8].

The McGill Quality of Life Questionnaire-Reviewed [9], improved the MQOL version, addressing issues that arose during the use of MQOL over the years. The results provided a well-adjusted measurement structure and expected correlations between each subscale of the MQOL-R and MQOL single item scale (SIS). MQOL-R has strong psychometric properties, and it has been widely used in palliative care in both clinical and research for the life quality at the end of life assessment. It has subscales measuring the four relevant domains: physical, psychological, existential/spiritual, and social. It evaluates the physical condition's impact on the quality of life, rather than on the intensity of symptoms. In contrast, most other quality life assessment tools at the end of life do not include the existential/spiritual domain, have a primary focus on physical symptoms, or have many more items. An additional advantage of the MQOL-R is that it takes approximately 5–10 min to complete a self-administered in paper and pencil or online format [9]. Considering that the area of palliative care is in rapid development, and that lack of appropriate instruments to assess QOL, this motivated us to translate and adapt of the MQOL-R to the Brazilian Portuguese (BrP) within its linguistic and sociocultural context.

Thus, we conduct the present study to examine the psychometric reliability of the translated MQOL-R for the Brazilian population [10]. (I) We evaluated the content validity and face validity by semantic equivalence, the comparison of items by experts, and a sub-sample of the target population to assess the cross-cultural, adapted from the English version of the Brazilian Portuguese (BrP)-MQOL-R. (II) We examined the internal consistency, criteria validity, factor structure, and construct validity of the MQOL-R translated instrument. (III) We assessed the convergence validity by the correlation of the MQOL-R with relevant correlates for the quality of life, such as the Karnofsky Performance Scale (KPS) and pain levels reported on the Numerical Pain Scale (NPS 0–10). (IV) We evaluated the criterion validity by the ability of MQOL-R to discriminate between subjects whose performance in the KPS equal to or lower than 30% those with a KPS higher than 30%.

## Methods

**Fig. 1:**
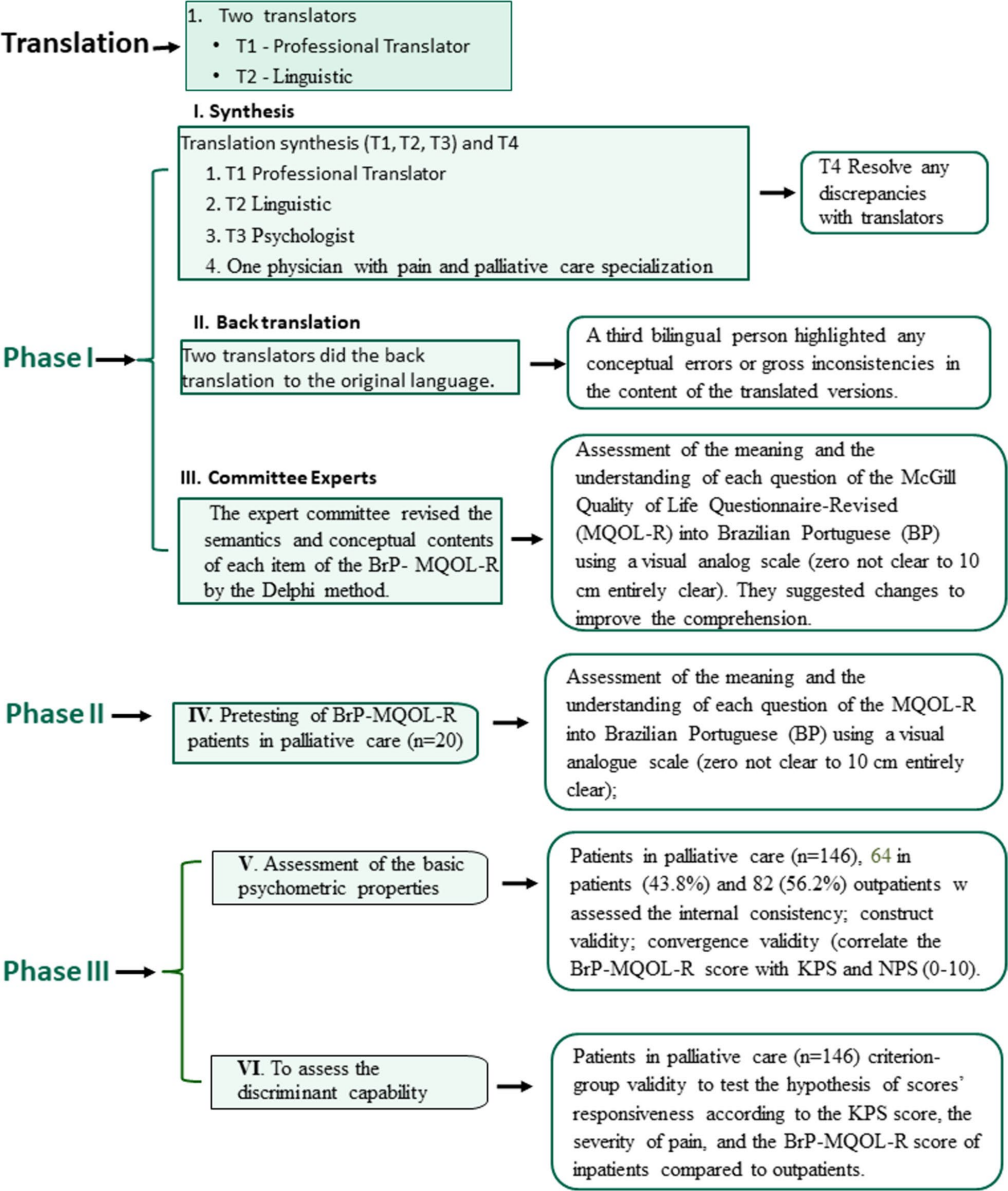
Flow of the multiple standardized phases of the study. Abbreviations: Karnofsky Performance Status Scale (KPS); Numerical Pain Scale (NPS0-10)

The protocol of this cross-sectional study was approved by the Ethics Committee Board of the Hospital de Clínicas de Porto Alegre, Brazil (protocol no 2019–0207). All subjects gave their written formal consent for participation or their caregivers. Figure 1 presents the flow of the standardized phases of the study.

### Phase I. Translation, synthesis and back translation and consensus of experts assessed the content and face validity

Previously published guidelines carried out the procedures for the translation and cross-cultural adaptation of the MQOL-R to Brazilian Portuguese [10–13]. We follow the recommended practices by the Health Measurement Consensus guideline terminology (COSMIN) for assessing the content validity for health‐related Patients. According to the COSMIN, evaluating the content validity for health‐related Patient Reported Outcome Measures (PROMS) is categorized into three broad domains: Reliability, containing the Measurement and assessment of the conceptual semantics content of each item [14]. The procedures for assessing the semantics and conceptual content of each item of the MQOL-R [14] were through the Delphi method [15]. The McGill Quality of Life Questionnaire-Revised (MQOL-R) validated for Brazilian Portuguese is presented in Additional file 1.

### Phase II. Pretesting of BrP-MQOL-R in a pilot study

Twenty patients assessed the comprehension of the item of BrP-MQOL-R. Among them, nine were inpatients, and 11 were women. The median age was 59.50 [interquartile ranges IQR) (IQR 25–75 = 46.5; 73.75)] and the median of formal schooling was 8 years (IQR 25–75 = 5; 11), respectively. They evaluated the meaning of the translated questions and the layout of the pre-final version of the BrP-MQOL-R and assessed each item's comprehension using a 10 cm visual analog scale (VAS; 0 completely incompressible to 10 cm entirely clear). The median of comprehension of all items was 8.70 (IQR 25–75 = 7.56; 10).

### Phase III. Assessment of psychometric properties and the validity of the final version of the BrP-MQOL-R

A total of 157 patients over 18 years old at the Pain and Palliative Medicine Service of “Hospital de Clínicas de Porto Alegre, Brazil, from March 2019 to December 2019. Sixty-four in patients (43.8%) and 82 (56.2%) outpatients. Patients illiterate and those with cognitive impairment that prevented them from answering questions or communicating were excluded. Data were obtained by trained evaluators using a standardized questionnaire, the MQOL-R, the Performance de Karnofsky Scale, and a Numerical Pain Scale (NPS 0–10).

### Self-report variables

NPS (0–10) was used to measure pain intensity, ranging from 0 (no pain) to 10 (the worst pain possible). They answered their pain level most of the time in the last 24 h and the pain score after taking pain medication.

The Karnofsky Performance Status Scale (KPS) was used to quantifying functional status. The KPS is an 11-point rating scale that ranges from normal functioning (100) to dead (0). The KPS of < 30 the patients unable to perform these activities with or without assistance [16].

The McGill Quality of Life Questionnaire-Revised (MQOL-R) consists of 14 items divided into four domains: The Single-Item Scale (SIS), physical symptoms (three items), feelings and thoughts (seven items), and social (three items). The overall scale has good internal reliability (α = 0.94) [10].

### Statistical analysis

We conducted descriptive statistics to examine the underlying assumptions of normality for all variables of interest. The Cronbach’s alpha and Spearman-Brown were used for the assessment of the MQOL-R’s reliability. For the BrP-MQOL-R, the maximum likelihood factor analyses with oblique rotation were conducted. We checked the scale's internal structure using confirmatory factor analysis (CFA) and establishing its reliability and validity. Items with a loading equal to or higher than 0.4 were retained to be considered relevant [17]. Factors that win with eigenvalues greater than one were also excluded. Convergent validity was evaluated by Pearson’s correlation coefficient between BrP-MQOL-R total scores, subscales, and the SIS measuring overall quality of life with scores on NPS (0–10) and the KPS scale. The non-parametric receiver operating characteristics (ROC) analyses, with the exact binomial of the area under the curve (AUCs) with 95% confidence intervals (CI), is presented. We calculated the standard errors (SEs) by Hanley’s method [18]. The cutoff values with the highest Youden index, with 90% sensitivity and 100% specificity, are presented for the BrP-MQOL-R with a ROC AUC 0.70. Finally, a stratified-by-sex analysis was used to assess the correlation between age, education level, if they were hospitalized when they answered the MQOL-R (Yes/No), and the scores of the dependent variable MQOL-R. We employed regression analysis with a stepwise forward technique. The prior sample size was estimated a priori based on the number of volunteers' ratio to the number of items. In this case, the MQOL-R has 14 questions. Based on this criterion, we needed 140 volunteers. Considering potential loss by insufficient data, we increased the sample size by 10% [10]. For all statistical analyses, significance was set at P < 0.05. The analysis used SPSS version 24.0 (IBM, Armonk, NY, USA), and the CFA was conducted by means of SPSS. AMOS. Version 24.0 (IBM, Armonk, NY, USA).

## Results

### Phase III: assessment of psychometric properties and the validity of the final version of the Validation study

#### Sample characteristics

The demographic data and clinical characteristics are presented in Table 1. There was a proportionate number of females and males in our sample, 53.4% and 46.6%, respectively. The mean scores of the BrP-MQOL-R for males were 5.69 (1.63) and for females 5.69 (2.20) (t = 2.75, P = 0.007], respectively. The mean score on the BrP-MQOL-R for the total sample was 6.09 (SD = 2.0). The median of all items was 6.17 [interquartile (IQR25-75) 4.67; 7.60].

**Table 1:** Sociodemographic and Clinical Characteristics of the Study Sample (n = 146)

Characteristic	Frequency (%) or mean (SD)	Median (IQ 25–75)
Sex		
Male/female	78 (53.4%)/68 (46.6%)	
Hospitalized		
Yes/no	64 (43.8%)/82 (56.2%)	
Education (years)	6.81 (4.17)	6 (0; 17)
	65.16 (14.04)	66 (58; 75)
Karnofsky performance status scale (KPS)	59.18 (21.38)	50 (40; 80)
Score ≤ 60/score > 61	42.77 (10.74)/80.79 (9.03)	50 (20; 60)/80 (70; 100)
Score NPS (0–10), most of the time in the last 24 h		
Cancer (yes/no)	4.4 (3.61)/4.74 (3.99)	5 (0; 10)/6 (0; 10)
Score on NPS (0–10) after use pain medication		
Cancer (yes/no)	2 (2.83)/2.21 (2.69)	0 (0; 10)/0 (0; 8)

Primary cancer clinical diagnosis	Frequency (%)	
Palliative care patients without cancer	44 (30)	
Lung carcinoma	15 (10)	
Head and neck cancer	15 (10)	
Melanoma	4 (3)	
Breast cancer	9 (6)	
Adenocarcinoma	6 (4)	
Esophagus cancer	9 (6)	
Prostate carcinoma	13 (9)	
Bowel cancer	1 (1)	
Colorectal cancer	5 (3)	
Stomach cancer	7 (5)	
Lymphoma	1 (1)	
Hepatoma	2 (1)	
Endometrial cancer	4 (3)	
Kidney cancer	2 (1)	
Bladder cancer	2 (1)	
Diagnosis to be confirmed	7 (5)	

### Psychometric properties of the MQOL-R- BrP

#### Internal consistency

The BrP-MQOL-R final 14-item had a satisfactory internal consistency (α = 0.85). The mean (SD) for all items of the scale was 6.09 [2].

The MQOL-R scale and subscales and the total result were scored by averaging across items. We checked whether the findings in subscales differ from one another; we conducted a one-way repeated-measures ANOVA. The data comply with the variance sphericity (Muychaly’s test: W = 0.89, P = 0.008). This result indicates that the results in the MQOL-R sub-scales differ from one another. The multiple comparison test by Bonferroni revealed that QOL in the subscale social [mean (standard deviation)] was highest in our sample [8.14, (1.87); P < 0.001 for all comparisons], followed by existential [6.36 (2.10) P < 0.001 for all comparisons], psychological [5.21 (2.60)], and physical [4.88 (2.01)]. Other comparisons returned no significant difference.

#### Construct validity: questionnaire item selection, structural validity and cross-cultural-validity

##### Confirmatory factor analysis of the MQOL-R

**Fig. 2:**
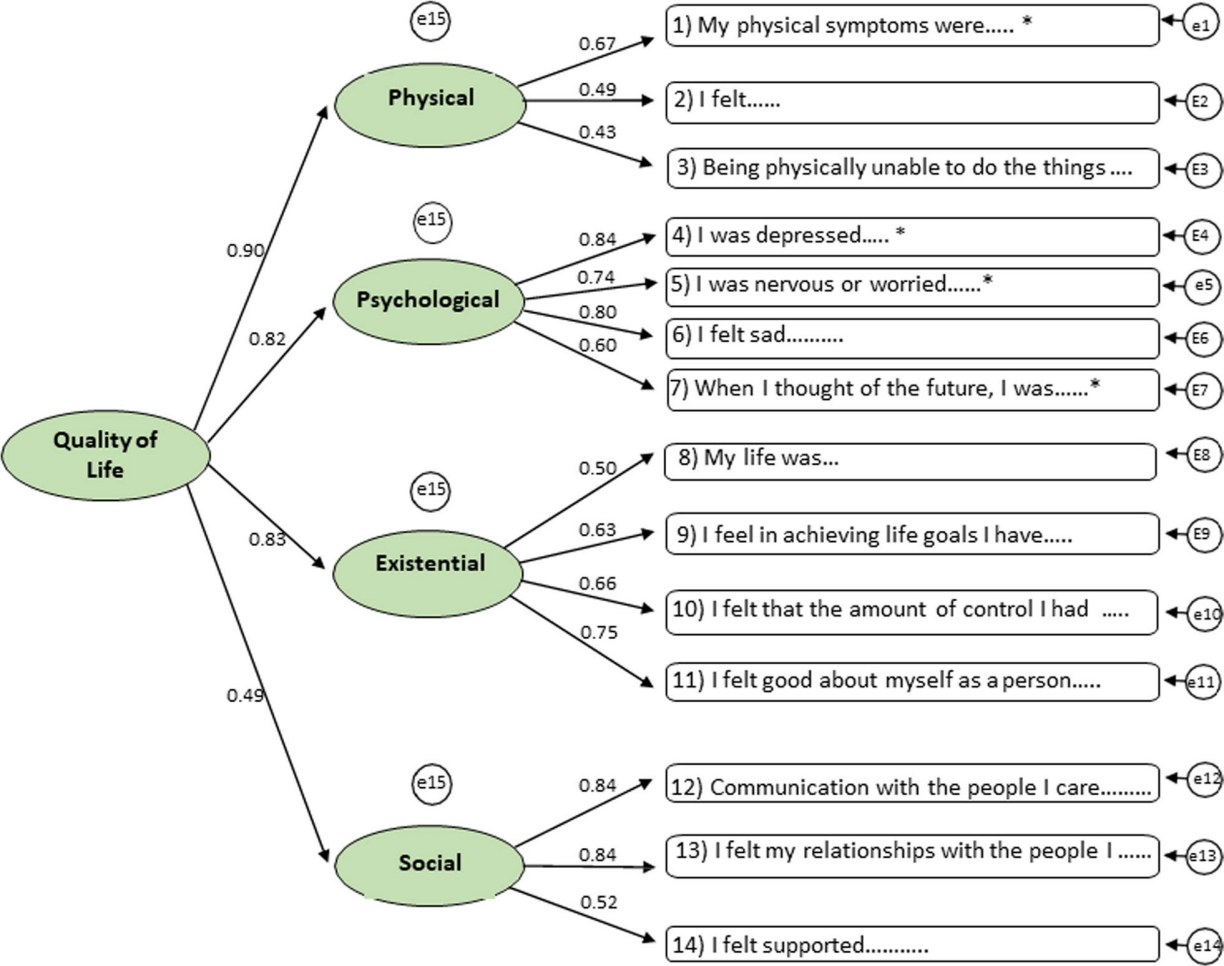
MQOL-R items and CFA for first order (subscale) and second order (overall QOL) latent factors. Factor loadings are standardized

We tested the internal structure of the MQOL-R using CFA, using the generalized least squares method. CFA revealed that all items were related to four specified factors, verifying the item's relationships and latent factors. Figure 2 shows the diagram and factor loading generated and presented in Table 2, the fit indices for this model. The analysis elicited adequate model goodness of fit (Table 2). The χ^2^ test (CMIN = 117.38; df = 73; p = 0.001) suggests insufficient fit, although this statistical tool is too restrictive and often points to rejecting a model with high samples involved. The chi-square/degree of freedom (CMIN/df = 1.608) reached a satisfactory value under 5. Following the strategy of presenting fit indices suggested by Hu and Bentler [19] if the root means the square error of approximation (RMSEA = 0.065; confidence interval 0.042–0.086) is 0.06 or below, and the standardized root-mean-square residual (SRMR) is 0.08 or below, thus, the model fitting is good. Comparative Fit Index (CFI = 0.934; RMSEA = 0.065; 95% CI range 0.042, 0.086). The revised model has the following fit-indices: PCLOSE = 0.131, Tucker-Lewis Index (TLI) rho 2 = 0.918, incremental fit index (IFI) delta 2 = 0.936. A second-order factor model was specified (Fig. 2) to support the derivation of an MQOL-R total score.

**Table 2:** Descriptive statistics, alpha coefficients for scores on the Brazil adaptation of the McGill Quality of Life Questionnaire-Items and the Total Score (n = 146)

Global QOL scores and items	Mean (SD)	Alfa de Cronbach
1. My physical symptoms were …. *	4.5 (3.6)	0.846
2. I felt (physically terrible–Physically well)	6.0 (2.9)	0.849
3. Being physically unable to do the things I wanted was…. *	4.1 (3.5)	0.855
4. I was depressed…..*	5.6 (3.8)	0.838
5. I was nervous or worried…..*	4.5 (3.7)	0.840
6. I felt sad….*	5.1 (4.0)	0.840
7. When I thought of the future…*	5.6 (3.8)	0.846
8. My life was……	6.2 (3.5)	0.853
9. I feel in achieving life goals I have: Made no progress…	6.4 (3.2)	0.848
10. I felt that the amount of control I had over my…	6.1 (3.6)	0.844
11. I felt good about myself as a person…	6.7 (3.2)	0.839
12. Communication with the people I care about …	7.7 (2.9)	0.849
13. I felt my relationships with the people I care about were…	8 (2.7)	0.847
14. I felt supported…	8.6 (2.4)	0.855
BrP-MQOL total score	6. 15 (1.96)	0.854

*QOL* quality of life

* Reverse-scored items

#### Convergence validity

The correlation between the BrP-MQOL-R total scale and subscale scores is displayed in Table 4. Convergence-related validity is also supported by significantly positively correlated with higher levels in the BrP-MQOL-R total scores and their subscales with both the KPS score and the SIS related to the quality of life. In contrast, the BrP-MQOL-R was conversely correlated with the pain scores in the NPS (0–10). Most of the time, patients with higher pain scores in the last 24 h and after use pain medication showed a lower score in the BrP-MQOL-R, or vice-versa.

The pain scores on NPS (0–10) in two conditions, the pain level on most of the time in the last 24 h and relive of pain score when you take pain medication. The mean (SD) on the KPS was 59.18 (21.38). The mean score (SD) in the SIS measuring overall quality of life was 6.5 (2.54). The mean (SD) on the question of their pain level on most of the time in the last 24 h was 4.50 (3.72), and after taking pain medication was 2.06 (2.78).

#### Responsiveness and criterion-group validity

The responsiveness of the BrP-MQOL-R can be seen by the mean (standard deviation) of the total score. Patients with KPS equal to or lower than 30% could be discriminated from those with scores on KPS higher than 30%; the score on the BrP-MQOL-R was 4.83 (1.77) vs. 6.36 (1.95) (P = 0.00), respectively. Also, the scores of the QOL scale and subscales tend to be higher in subjects with the best functional status. That is means that this tool has properties to capture differences between patients in palliative care with the worst performance of those who have better functional status. We assessed the criterion validity by the screening accuracy to discriminate patients with KPS equal to or lower than 30% (n = 25) those with scores on KPS higher than 30% (n = 121) by non-parametric receiver operating characteristics (ROC) analyses an area under the curve (AUC) = 0.71, sensitivity = 97% and specificity = 92%).

A regression analysis was used to assess if sex, hospitalization, formal education, and age could influence the score in the BrP-MQOL-R. The variables retained in the model were sex and the hospitalization at the time of assessment, the beta-coefficient was − 0.86 (95% CI; − 1.49 to − 0.23; P = 0.00) and 0.78 (95% CI; 0.15 to 1.42; P = 0.01), respectively. That is, females and, if they were at home at the time of the assessment, showed higher scores.

Separate regression analyses were performed to determine the global BrP-MQOL-R score and a combination of the BrP-MQOL-R subscales to predict the SIS. These models were adjusted by hospitalization at the time of assessment adjusted and sex. The total score predicted similar variance in the SIS (R^2^ adjusted = 0.36; β = 0.49, t = 4.50, p < 0.001) than those found in the MQOL-R subscales (R^2^ adjusted = 0.36). A combination of two subscales was significant in predicting the SIS: Physical (β = 0.25, t = 2.43, p < 0.01) and Existential (β = 0.21, t = 2.29, p = 0.02).

## Discussion

These results display data about the cross-cultural adapted to the English version of the BrP-MQOL-R. The process of translating and back translating the English BrP-MQOL-R to the Brazilian Portuguese translation was carried out stringently following established guidelines [10]. The set of questions of the BrP-MQOL-R presented satisfactory internal reliability with Cronbach's alpha coefficients higher than 0.85, likewise to the original English version. Our findings indicated an adequate construct validity and internal consistency of the BrP-MQOL-R translated and adapted to Brazilian Portuguese [10]. They also showed that the items with higher load are those related to social and psychosocial, and the lowest was found in the physical domains.

The content validity is evidenced by the high scores of the questionnaire items for readability, clarity, and comprehensiveness, as demonstrated by the scores on the visual analog scale in the assessment of the expert's committee consulted. Likewise, the result was found in a sample of patients in palliative care. This process yielded a Brazilian Portuguese version of MQOL-R semantically equivalent to the English language MQOL-R. Thus, the current version of the BrP-MQOL-R can be used without significant difficult in Portuguese-speaking populations. The test for internal consistency by Cronbach’s alpha indicates that either in the items and domains showed adequate consistency among their responses (see Tables 2, 3). These internal consistency coefficients by Cronbach's alpha are like them obtained original scale [8].

**Table 3:** Alpha coefficients for scores on the Brazil adaptation of the McGill Quality of Life Questionnaire-Revised and their subscales (n = 146)

Subscales	Mean (SD)	Alfa de Cronbach
BrP-MQOL-physical domain	4. 88 (2.43)	0.822
BrP-MQOL psychological domain	5. 22 (3.13)	0.817
BrP-MQOL existential domain	6. 36 (2.54)	0.816
BrP-MQOL social domain	8. 15 (2.25)	0.858
BrP-MQOL total score^$^	6. 15 (1.96)	0.740

^$^ BrP-MQOL-R summary score calculated by taking the mean of the subscale scores to give each domain equal weight

CFA of the BrP-MQOL-R using a variety of different goodness of-fit model measures indicate an adequate construct validity. Like the original version, the model shows the goodness of fit with four factors: Existential, Social, Psychological, and Physical. [9] The CFA demonstrated that all items of four factors showed a load factorial higher than 0.4. This result indicates that all elements of each factor converge to a common point to constitute a construct. Thus, our result confirms how well our analyzed variables represent the original constructs [9]. A strength of BrP-MQOL-R is items in each of the four subscales remain as proposed by Robin Cohen et al. [9] The CFA suggests that it is possible to maintain the original structure scale items in the BrP-MQOL-R. Also, the factor analysis supports using separate scores for each one of the four domains.

We found moderate correlations between several domains, indicating that one life domain experience is related to other domains. Further, to examine the convergence validity of BrP-QOL-R, we analyzed the strength of the relationship with the functional status by KPS score and in the SIS about the quality of life. All correlation among these factors showed correlations coefficients less than 0.5 (see Table 4). According to literature, the correlation for concurrent validity measure similar concepts could not exceed 0.7 [20]. This way, the KPS scores’ correlation, either with the BrP-MQOL-R and their domains, indicates convergent validity. These results showed that these are measuring aspects of the same construct but not in an identical way. The KPS evaluates the functional status at the end of life, such as the patient’s ability to carry on his everyday activity and work or his need for a specific custodial care amount dependence or constant medical care to continue alive. These simple criteria serve to measure the burden that the patient's care represents to his family or society and indirectly evaluate aspects of life quality. We used the same rationale related to the convergent validity to interpret the weak association of the MQOL-R score with an SIS QOL (r = 0.33). However, in this case, the converse correction among the QOL score and their subscales indicates that this tool and its subscales can identify the negative impact of pain on life quality. From the clinical perspective, they support improving educational programs to improve pain management to relieve patients' suffering in palliative care.

**Table 4:** Correlations among the MQOL-RBr total scores and their dominions, the single-item related to quality of life, functional status, and severity of pain (n = 146)

	(1)	(2)	(3)	(4)	(5)	(6)	(7)	(8)
MQOL-RBr physical dominion (1)	1							
MQOL-RBr psychological dominion (2)	.57**	1						
MQOL-RBr existential dominion (3)	.43**	.47**	1					
MQOL-RBr social dominion (4)	.28**	.30**	.43**	1				
MQOL-RBr total score (5)	.74**	.84**	.79**	.60**	1			
Single-item scale measuring overall quality of life (6)	.26**	.22**	.29**	.25^**^	.33**	1		
Karnowski Performance Scale (7)	.47**	.35**	.34**	.24**	.46**	.16*	1	
Score on NPT (0–10) after use pain medication (8)	− .37**	− .40**	− .20*	− .12	− .37**	− .27**	− .17*	1
Score NPS (0–10), most of the time in the last 24 h (9)	− .47**	− .37**	− .22**	− .20*	− .42**	− .27**	− .31**	− 0.18*

** Correlation is significant at the 0.01 level (2-tailed)

* Correlation is significant at the 0.05 level (2-tailed)

The relevance of these results is to evidence that the BrP-MQOL-R showed a sensibility identify the effect of factors that contribute to worst QOL either cancer or non-cancer patients. For example, the pain level, which is a specific aspect of healthcare, is a person-centered experience. In sum, these findings demonstrated that this tool validated and adapted to the Brazilian population is suitable as part of an assessment of "quality of life" in patients in palliative care. Another measure that showed the theoretical construct of the BrP-MQOL-R is the criterion-validity to differentiate those patients unable to perform their activities with or without assistance compared to those that need medical care but less than the distinguished group. Thus, this intensive process to establish the validity of the BrP-MQOL-R provided reliable support for its validity in more depth. Thereby, we can offer the Brazilian population an instrument to assess the quality of life" in palliative care adequately adapted. This is important to clinical and for research from a transcultural perspective. Notably, it would be a useful tool to evaluate how the impact of support pharmacological and non-pharmacological in palliative care in different cultures. Mainly because in patients under palliative care, the illnesses are in progress, and healthcare takes on an increasingly important role day by day in these people’s life. Hence, the quality of life should be the most target in the care of these patients.

In the present study, males were associated with the worst quality of life compared to females. Accordingly, prior research investigating sex differences in aggressiveness of end-of-life care preferences [21] and women are less likely to prefer life-sustaining technology and other aggressive treatments. Also, they are more likely to give do-not-resuscitate orders to have a dignified death [21]. While another survey found that among patients with advanced cancer, women were more likely than men to recognize that their disease was incurable and at an advanced stage and report having discussed life expectancy with their oncologist [22]. Another result that evidenced the discriminatory properties of the validated scale was identifying the worst quality of life of patients in the hospital compared to patients in palliative care at home. This finding is plausible and supported by earlier surveys conducted in the United States (US), the UK, and the Netherlands, which reported that the quality of the end of life in hospitals was not satisfactory [23–25].

The main limitations of this study should be addressed. First, the test–retest was not performed. However, it is important to realize that the reliability of the test–retest gives more reliable results when a patient's health status is stable at both times of the test [26]. In the context of palliative care, the clinical status changes faster sometimes in hours or in a few days at the way that this measure would be less reliable. Second, the study is limited by the nonrandom selection of patients recruited in palliative care service at a university hospital. Hence, selection bias is possible, and it is uncertain whether these findings can be extrapolated to patients receiving treatment in hospitals without palliative care. However, it is noteworthy that our results are consistent with findings observed in the original English language version, which involved a variety of a representative national sample [27]. Third, the study is based on self-report measures. Thus, the comprehension of items content of the assessment instruments may have implications for the internal validity of the survey. Finally, longitudinal studies are required with a more significant number of clinical samples.

## Conclusion

This study provided evidence for the validity, reliability and demonstrated that the psychometric properties of the BrP-MQOL are satisfactory. Also, it showed adequate discriminate properties being sensitive to detecting the general conditions of patients with a terminal disease involving patients in palliative care in Portuguese-speaking countries. In sum, they suggest that this scale represents a valuable instrument for use in scientific studies and in the clinical setting involving patients in palliative care (Additional file 1).

## Supplementary information

**Additional file 1**. The McGill Quality of Life Questionnaire-Revised (MQOL-R) validated for Brazilian Portuguese

## Data Availability

The datasets used and/or analysed during the current study are available from the corresponding author on reasonable request.

## Abbreviations

AUC: Area under the curve
BrP-MQOL-R: Brazilian Portuguese McGill Quality of Life Questionnaire-Reviewed
CFA: Confirmatory factor analysis
CI: Confidence interval
CMIN: Chi square
COSMIN: Consensus Guideline Terminology
df: Degree of freedom
HCPA: Hospital de Clinicas de Porto Alegre
IFI: Incremental fit index
IQR: Interquartile ranges
KPS: Karnofski performance score
MQOL-R: McGill Quality of Life Questionnaire-Reviewed
NPS: Numeric Pain Scale
PC: Palliative care
PROMS: Patients Reported Outcome Measures
QOL: Quality of life
ROC: Receiver operator characteristics
RMSEA: Root means the square error of approximation
SD: Standard deviation
SEs: Standard errors
SIS: Single Item Scale
SRMR: Standardized root-mean-square residual
TLI: Tucker Lewis index
UCACUE: Universidad Católica de Cuenca
UFRGS: Universidade Federal de Rio Grande do Sul
UK: United Kingdom
US: United States
VAS: Visual Analogue Scale
