# Supplementary material for: The McGill Quality of Life Questionnaire-Revised (MQOL-R). Psychometric properties and validation of a Brazilian version on palliative care patients: a cross-sectional study
Source: Health Qual Life Outcomes. 2020 Nov 14;18:368. doi: 10.1186/s12955-020-01621-8 (PMC7666518; doi:10.1186/s12955-020-01621-8)
Supplement: Supplementary file 1 — Additional file 1. The McGill Quality of Life Questionnaire-Revised (MQOL-R) validated for Brazilian Portuguese [file 12955_2020_1621_MOESM1_ESM.pdf]

*© 2017 S. Robin Cohen*

*B2019 - Caumo et all. - Adapted to Brazilian Português*
*E-mail: wcaumo@hcpa.educ.br*

# *McGill QUALITY OF LIFE QUESTIONNAIRE Revised©*
# *QUESTIONÁRIO DE QUALIDADE DE VIDA DE MCGILL Revisado[B]*
# *Adaptado para o Português do Brasil*

Idade: Escolaridade: Sexo: F ( ) M ( )

**INSTRUÇÕES**

**As perguntas deste questionário começam com uma afirmação com possibilidade de respostas extremas:**

**0 (ausência do sintoma) e 10 (sintoma com intensidade máxima).**

**Por favor, circule o número, entre 0 e 10, que melhor representa o que você está sentindo.**

**Não há respostas certas ou erradas. Por favor, responda com honestidade.**

Estou com fome

| | | | | | | | | | | | | |
|---|---|---|---|---|---|---|---|---|---|---|---|---|
| Nenhuma fome | 0 | 1 | 2 | 3 | 4 | 5 | 6 | 7 | 8 | 9 | 10 | Muitíssima fome |

EXEMPLO

- **Se você não está com fome, circule 0.**
- **Se você está pouca fome você pode circular 1, 2 ou 3.**
- **Se você estiver com fome moderada você pode circular 4, 5 ou 6.**
- **Se você está com muita fome você pode circular 7, 8 ou 9.**
- **Se você está com muitíssima fome, circule 10.**

COMECE AQUI:

RESPONDA TODAS AS PERGUNTAS

CONSIDERE COMO VOCE SE SENTIU NOS ÚLTIMOS DOIS DIAS.

*© 2017 S. Robin Cohen*

*B2019 - Caumo et all. - Adapted to Brazilian Português*
*E-mail: wcaumo@hcpa.educ.br*

## PARTE A - Qualidade de vida global

Nos últimos dois dias, considerando os aspectos físicos, emocionais, sociais, espirituais e financeiros a minha qualidade de vida tem sido:

| | | | | | | | | | | | | |
|---|---|---|---|---|---|---|---|---|---|---|---|---|
| Muito ruim | 0 | 1 | 2 | 3 | 4 | 5 | 6 | 7 | 8 | 9 | 10 | Excelente |

## PARTE B – SINTOMAS FÍSICOS

1. Nos últimos dois dias, meus sintomas físicos (como dor, náusea, cansaço e outros) causaram*

| | | | | | | | | | | | | |
|---|---|---|---|---|---|---|---|---|---|---|---|---|
| Nenhum problema | 0 | 1 | 2 | 3 | 4 | 5 | 6 | 7 | 8 | 9 | 10 | Graves problemas |

*Se houve sintomas físicos que foram um problema

Por favor, liste-os (por favor, escreva com clareza)

__________________________________________________

__________________________________________________

__________________________________________________

2 . Nos últimos dois dias, eu me senti fisicamente:

| | | | | | | | | | | | | |
|---|---|---|---|---|---|---|---|---|---|---|---|---|
| Péssimo | 0 | 1 | 2 | 3 | 4 | 5 | 6 | 7 | 8 | 9 | 10 | Ótimo |

3. Nos últimos dois dias, me senti incapaz para fazer as coisas que eu queria fazer:

| | | | | | | | | | | | | |
|---|---|---|---|---|---|---|---|---|---|---|---|---|
| De modo Nenhum | 0 | 1 | 2 | 3 | 4 | 5 | 6 | 7 | 8 | 9 | 10 | Completamente |

4. Nos últimos dois dias, estava deprimido(a):

| | | | | | | | | | | | | |
|---|---|---|---|---|---|---|---|---|---|---|---|---|
| De modo Nenhum | 0 | 1 | 2 | 3 | 4 | 5 | 6 | 7 | 8 | 9 | 10 | Extremamente |

5. Nos últimos dois dias, fiquei ansioso(a) ou preocupado(a):

| | | | | | | | | | | | | |
|---|---|---|---|---|---|---|---|---|---|---|---|---|
| De modo nenhum | 0 | 1 | 2 | 3 | 4 | 5 | 6 | 7 | 8 | 9 | 10 | Extremamente |

6. Nos últimos dois dias, me senti triste:

| | | | | | | | | | | | | |
|---|---|---|---|---|---|---|---|---|---|---|---|---|
| Nunca | 0 | 1 | 2 | 3 | 4 | 5 | 6 | 7 | 8 | 9 | 10 | Sempre |

7. Nos últimos dois dias, quando pensei sobre meu futuro percebi que estava:

| | | | | | | | | | | | | |
|---|---|---|---|---|---|---|---|---|---|---|---|---|
| Sem medo | 0 | 1 | 2 | 3 | 4 | 5 | 6 | 7 | 8 | 9 | 10 | Apavorado (a) |

8. Nos últimos dois dias, a minha vida estava:

| | | | | | | | | | | | | |
|---|---|---|---|---|---|---|---|---|---|---|---|---|
| Sem sentido e sem significado | 0 | 1 | 2 | 3 | 4 | 5 | 6 | 7 | 8 | 9 | 10 | Com muito sentido e significado |

9. Quando eu penso na minha vida e nos objetivos que tinha eu sinto que:

| | | | | | | | | | | | | |
|---|---|---|---|---|---|---|---|---|---|---|---|---|
| Nao progredi em nada | 0 | 1 | 2 | 3 | 4 | 5 | 6 | 7 | 8 | 9 | 10 | Progredi em tudo |

10. Nos últimos dois dias, senti que eu tive controle sobre a minha vida

| | | | | | | | | | | | | |
|---|---|---|---|---|---|---|---|---|---|---|---|---|
| Nenhum | 0 | 1 | 2 | 3 | 4 | 5 | 6 | 7 | 8 | 9 | 10 | Completamente |

11. Nos últimos dois dias, me senti bem comigo mesmo.

| | | | | | | | | | | | | |
|---|---|---|---|---|---|---|---|---|---|---|---|---|
| Discordo completamente | 0 | 1 | 2 | 3 | 4 | 5 | 6 | 7 | 8 | 9 | 10 | Concordo completamente |

## PARTE D –Social

12. Nos últimos dois dias, a comunicação com as pessoas de quem gosto foi

| | | | | | | | | | | | | |
|---|---|---|---|---|---|---|---|---|---|---|---|---|
| Muito difícil | 0 | 1 | 2 | 3 | 4 | 5 | 6 | 7 | 8 | 9 | 10 | Muito fácil |

13. Nos últimos dois dias, senti que o meu relacionamento com as pessoas de quem gosto tem sido

| | | | | | | | | | | | | |
|---|---|---|---|---|---|---|---|---|---|---|---|---|
| Mais distante do que gostaria | 0 | 1 | 2 | 3 | 4 | 5 | 6 | 7 | 8 | 9 | 10 | Muito próximo |

14. Nos últimos dois, dias me senti apoiado:

| | | | | | | | | | | | | |
|---|---|---|---|---|---|---|---|---|---|---|---|---|
| De modo nenhum | 0 | 1 | 2 | 3 | 4 | 5 | 6 | 7 | 8 | 9 | 10 | Completamente |
